# Systematic care for caregivers of people with dementia in the ambulatory mental health service: designing a multicentre, cluster, randomized, controlled trial

**DOI:** 10.1186/1471-2318-9-21

**Published:** 2009-06-07

**Authors:** Anouk Spijker, Frans Verhey, Maud Graff, Richard Grol, Eddy Adang, Hub Wollersheim, Myrra Vernooij-Dassen

**Affiliations:** 1 Scientific Institute for Quality of Healthcare (IQ Healthcare), Radboud University Nijmegen Medical Centre, Nijmegen, The Netherlands; 2 Alzheimer Centre Radboud University, Radboud University Nijmegen Medical Centre, Nijmegen, The Netherlands; 3 Department of Psychiatry/Alzheimer Centre Maastricht, University Hospital of Maastricht, Maastricht, The Netherlands; 4 Department of Epidemiology, Biostatistics and HTA, Radboud University Nijmegen Medical Centre, Nijmegen, The Netherlands; 5 Department of primary care and nursing home medicine, Radboud University Nijmegen Medical Centre, Nijmegen, The Netherlands; 6 Kalorama Foundation, Beek-Ubbergen, The Netherlands

## Abstract

**Background:**

Care for people with dementia and their informal caregivers is a challenging aim in healthcare. There is an urgent need for cost-effective support programs that prevent informal caregivers of people with dementia from becoming overburdened, which might result in a delay or decrease of patient institutionalization. For this reason, we have developed the Systematic Care Program for Dementia (SCPD). The SCPD consists of an assessment of caregiver's sense of competence and suggestions on how to deal with competence deficiencies. The efficiency of the SCPD will be evaluated in our study.

**Methods and design:**

In our ongoing, cluster, randomized, single-blind, controlled trial, the participants in six mental health services in four regions of the Netherlands have been randomized per service. Professionals of the ambulatory mental health services (psychologists and social psychiatric nurses) have been randomly allocated to either the intervention group or the control group. The study population consists of community-dwelling people with dementia and their informal caregivers (patient-caregiver dyads) coming into the health service. The dyads have been clustered to the professionals. The primary outcome measure is the patient's admission to a nursing home or home for the elderly at 12 months of follow-up. This measure is the most important variable for estimating cost differences between the intervention group and the control group. The secondary outcome measure is the quality of the patient's and caregiver's lives.

**Discussion:**

A novelty in the SCPD is the pro-active and systematic approach. The focus on the caregiver's sense of competence is relevant to economical healthcare, since this sense of competence is an important determinant of delay of institutionalization of people with dementia. The SCPD might be able to facilitate this with a relatively small cost investment for caregivers' support, which could result in a major decrease in costs in the management of dementia. Implementation on a national level will be started if the SCPD proves to be efficient.

**Trial Registration:**

NCT00147693

## Background

Estimates state that the rapidly aging western European population will peak at about 2040 [1]. An aging population demands more healthcare and challenges the healthcare budget. Two-thirds of the people with dementia (also referred to as "patients" in this study protocol) are cared for at home [2]. Care at home is often intensive and burdensome. Informal caregivers of these patients carry a greater burden than informal caregivers of other chronically ill people [3], and they are at a greater risk of depression [4-6]. The institutionalized care of people with dementia is one of the three most expensive areas of healthcare [7,8]. Furthermore, as a result of the growing elderly population, shortages within institutional care are expected. The resulting budgetary constraint necessitates the exploration of temporary alternatives, such as postponement of institutionalization and care at home. Without unpaid informal caregivers, the costs of professional care at home would double [9]. Support is needed to prevent informal caregivers becoming overburdened and depressed. An informal caregiver's sense of being capable of giving care is a strong determinant of delaying institutionalization [10]. Contemporary policies have therefore been designed to shape conditions to support caring for people with dementia at home and to minimize the risk of depression for informal caregivers.

### Usual ambulatory mental healthcare

In the usual healthcare, in the Netherlands, the problems of informal caregivers often remain invisible until a crisis occurs. This happens partly because informal caregivers pay scant attention to their own problems, and professionals may not know how to support informal caregivers pro-actively [11]. When informal caregivers have become involved in care provided by the ambulatory mental health services (hereafter referred to as the "health services"), they are rarely screened in a structured manner for the problems they may encounter. There is, for example, no systematic screening for the care burden or depressive symptoms. Informal caregivers suffering from depressive symptoms are either treated inadequately or not at all [12,13]. Moreover, the available support of the health service varies from support groups for informal caregivers to case management for active support and organization of the care needed [14]. One of the reasons for this unsystematic care for informal caregivers is the lack of a national guideline for the health services for patients with dementia and the insufficient recognition of informal caregivers as part of the system surrounding these patients. This fragmented care is reflected in the different functions of the health services. The health services set their own standards for the care of patients and their caregivers. This service is provided in collaboration and concurrence with other regional providers.

### Effective support programs for caregivers of people with dementia

Usual care offers many opportunities to support informal caregivers that remain unused because of the late detection and the ad hoc identification and management of possible caregiver problems [12-15]. Several support programs for these caregivers have been developed, some of which have proven to be effective [16]. Most programs aim at reducing the caregiver's burden or enhancing feelings of competence in caring, and their purpose is to delay patient institutionalization. Dutch examples of these programs are the Family Support Program [17,18], the Meeting Centres Support Program [19], and the Community Occupational Therapy Intervention for patients with dementia and their informal caregivers [20,21]. These proven effective support programs found successively positive changes in caregiver depressive symptoms [22], patients' problem behavior and caregiver distress as related to patients' problem behavior [23-26], sense of competence and feelings of competence being a female caregiver sharing the same household with the patient [18,27], as compared to changes in the controls. Moreover, proven effective support programs found a positive influence of patients' severity of dementia on delay of nursing home placement, as compared to the control groups [28,29]. As part of the multicomponent intervention behavioral and cognitive strategies were used to train caregivers and patients in the use of aids to compensate for cognitive decline and to cope with distressing behavior. Caregiving intervention studies appeared effective in improving caregiver psychological health and quality of life [19,30-33] as well as patients' quality of life [34].

### The Systematic Care Program for Dementia

We have transformed the Family Support Program into a Systematic Care Program for Dementia (SCPD) that can be used in the first consultation of a professional with a patient and his/her informal caregiver (a patient-caregiver dyad) entering the health service. The SCPD consists of an assessment of the caregiver's sense of competence and suggestions on how to deal with competence deficiencies. The SCPD has been chosen because of its potential to help diagnose and treat problems systematically and to cover a wide range of individual problems. The SCPD is flexible in connecting pro-active interventions to individual problems. Moreover, it is also connected to the positive effects found in our previous study [18,35]. This program has been designed to fulfill the urgent need for effective and cost-effective support programs that can prevent overburdening the informal caregiver, which might result in a delay or decrease of patient institutionalization.

### Objectives

The objective of this study protocol is to describe the design of a trial to determine both the effectiveness and the efficiency of the SCPD in comparison to regular ambulatory mental healthcare. The aim of the program is to delay the institutionalization of the patient with dementia and to improve the health-related quality of life of both the patient and the informal caregiver.

### Hypothesis

We expect a delay of patient institutionalization in the intervention group as compared to controls at 12 months follow-up. In addition, we expect that time to institutionalization will be longer in the intervention group as compared to the control group.

## Methods and design

### Study design and setting

**Figure 1:**
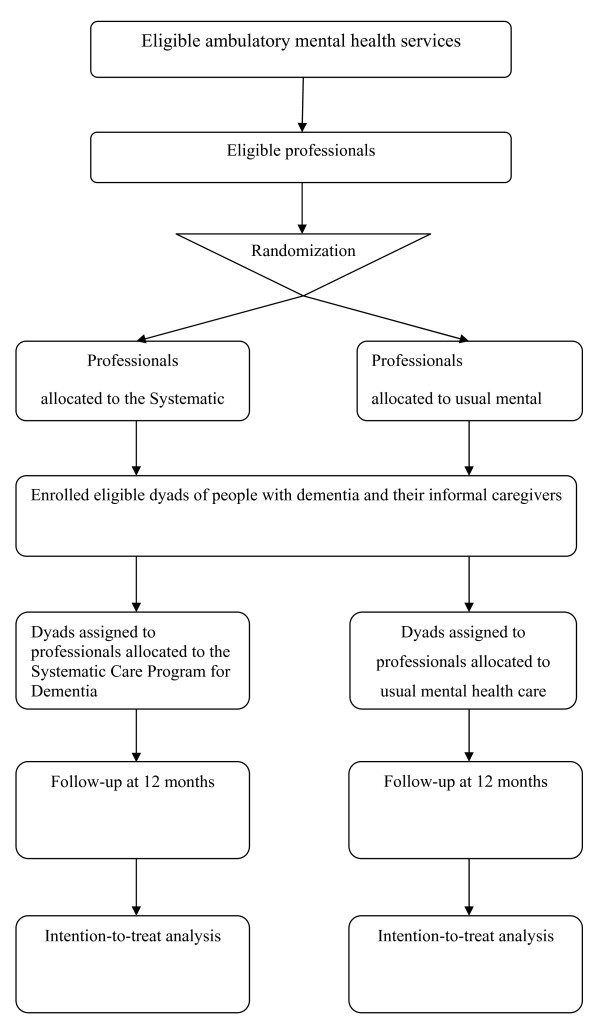
**Flow of the participants through the trial**.

The study design is a single-blind, multicentre, cluster, randomized, controlled trial. From September 2005 to February 2006, the research assistant enlisted and randomized professionals (psychologists and social psychiatric nurses), initially from four health services, either to the intervention group or the control group. One service dropped out because of the interference with another clinical trial. In order to enroll patients in due time three other mental health care services were included. Altogether, professionals from six health services and four regions were randomized to either the intervention group or the control group. This setback in recruiting patient-caregiver dyads prolonged the inclusion period by 4 months to a total of 17 months. The follow-up period has been set at 1 year. One year proved long enough for us to find significant effects in our previous study [36]. Figure 1 presents the flow of the participants through the trial at each randomization procedure.

The study cannot be double-blinded because all the professionals involved are aware of the treatment allocation. To prevent contamination, the intervention group have been asked to keep the study intervention secret and to inform neither professionals in the control group nor other colleagues nor field relations. The patient-caregiver dyads have been blinded to the group allocation of the professionals to whom they have been assigned. The intervention group will be trained to integrate the SCPD in their treatment method. Moreover, we assume that professionals will not be interested in telling patient-caregiver dyads that they are using a new or presumably better treatment method.

### Eligibility criteria

Health services were considered eligible if they could enroll clients with suspected dementia or with dementia. Furthermore, they had to be sure that they could recruit enough patient-caregiver dyads.

Professionals recruited by the health services were considered eligible if they treated at least four of the patient-caregiver dyads each year (see the section *Power calculations*). This is the minimum number required for reasons of continuity and routine. Moreover, this minimum is necessary so that the intervention professionals can change their daily routine and become familiar with the SCPD method.

The eligibility criteria for the dyads were:

1. The patient was referred to and entering the health service with the suspicion of dementia.

2. The patient lives in the community.

3. The patient has an informal caregiver living in the community.

4. The informal caregiver visits the patient at least twice a week.

5. The informal caregiver is willing to participate and gives written informed consent.

The exclusion criteria were:

1. The patient has no informal caregiver.

2. The informal caregiver is a client of the health service her/himself.

3. The informal caregiver is seriously ill and unable to participate in the study.

4. The informal caregiver does not speak Dutch fluently.

### Informed consent procedure

The informed consent procedure consisted of several steps. First, a psychologist or social psychiatric nurse provided written information for the informal caregiver. An informal caregiver who gave verbal consent and accepted the conditions was included in the next step. In this phase, the research assistant contacted the informal caregiver and made an appointment for the baseline interview, explained the assessment procedure, and answered questions. The informal caregiver was informed about the randomization procedure. After having given verbal consent, s/he received written confirmation of willingness to participate and the baseline questionnaire by post. An informal caregiver who was still willing to participate signed and returned the consent form and was included in the study.

### Treatment in the intervention group: the Systematic Care Program for Dementia

The experimental intervention (training in the SCPD and its subsequent use) is based on the "family support model" as developed by Gruenberg [37] and Bengtson and Kuypers [17]. Its purpose is to strengthen the caregiver's competence and sense of competence. Basically, the SCPD consists of an assessment of the caregiver's sense of competence and depressive symptoms, and suggestions about how to deal with deficiencies. It can begin in the first consultation between a professional and a patient-caregiver dyad.

**Figure 2:**
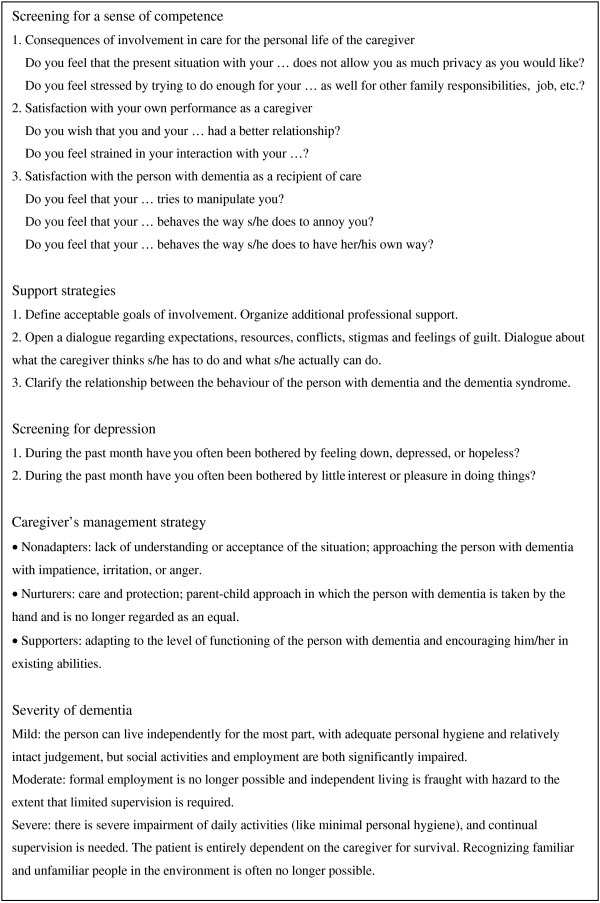
**Screening tool for the Systematic Care Program for Dementia**.

The SCPD can be divided into three stages:

1. *Screening*. Professionals screen the caregiver's sense of competence and depressive symptoms with the SCPD screening tool (inventory and interpretation) as presented in Figure 2. This means that professionals provide data about the Short Sense of Competence Questionnaire [38], depressive symptoms [39], and caregiver type [40]. They also provide their observations on the severity of dementia according to the *Diagnostic and statistical manual of mental disorders, 3^*rd *^text revision *(DSM-III-TR) [41].

2. *Psychosocial support*. The SCPD is flexible in connecting pro-active interventions to individual problems. Professionals use strategies aimed to support caregivers; for example, instructions on how to deal with the patient's behavioral problems. The clarification of the relation between the disease and the patient's problematic behavior is a SCPD support strategy. One goal might be that the caregiver will not take difficult behavior personally, which can diminish mutual negative feelings considerably. Professionals provide data about their support and interventions (actions) during and after each contact with the patient-caregiver dyad.

3. *Transfer to regular healthcare*. Along with psychosocial support, professionals might negotiate or organize respite care, which is like home care or day care. If the screening for caregiver depression gives cause for further screening for clinical depression, the professional may also refer the caregiver or start treatment. Professionals provide data about the organization and management of care if the case is transferred to other institutions or professionals.

### Training in the Systematic Care Program for Dementia

**Figure 3:**
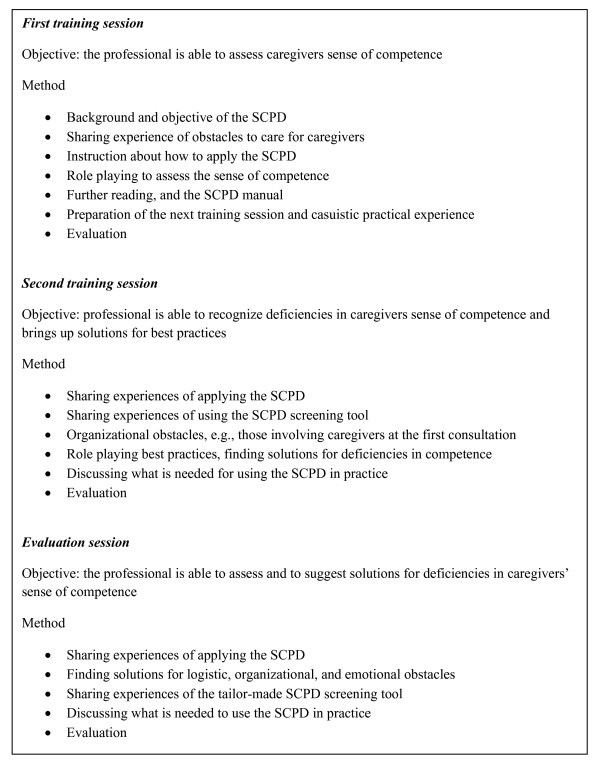
**Training program for the Systematic Care Program for Dementia**.

The training to teach professionals to use the SCPD consists of three sessions of 2 hours each. One meeting is for explaining the program, and two meetings are for the evaluation of the use of the program and for preparing suggestions on how to hand over the responsibility for care after the health service's work is completed. Figure 3 summarizes the objectives and methods used in the three training sessions. Several aids have been developed to facilitate the use of the SCPD:

1. *The SCPD screening tool*. The questions for the screening a caregiver's sense of competence and depressive symptoms are printed on a handy plasticized pocket card.

2. *The SCPD manual*. The manual consists of the items to be discussed during the training sessions (i.e., background information and methods), and some supporting literature has been added.

3. *The starter package and action list*. Several forms have been developed for the requested data. The starter package contains the forms that professionals need to gather these data. The action list contains 60 possible intervening and supportive actions that professionals might undertake as a result of the screening. They are divided into nine categories: intake, diagnostics, psychoeducation, psychosocial care, medical care, how to hand over care, legal care, case management, and crisis management. The list was developed in collaboration with a staff member and a social psychiatrist from each of the original three participating health services. Each person involved listed potential intervention and support actions. Repeatedly mentioned actions were included in the action list. Consensus for including actions on this list was reached.

### Treatment in the control group: usual ambulatory mental healthcare

Professionals randomized in the control group will continue their treatment for patient-caregiver dyads as usual. During the study period they will not receive the training in the SCPD. Usual ambulatory mental healthcare is characterized by late detection of caregiver problems and unsystematic support that differs among the health services [11-14].

### Data collection at baseline and follow-up measurements

During the informed consent procedure, caregivers were asked to complete the baseline questionnaire. The research assistant and three trained interviewers collected baseline data and will collect follow-up data. If the caregiver cannot answer the questionnaire independently, assistance from the research assistant or the interviewer will be offered. Measurements take place at baseline (T_0_) and 3 (T_1_), 6 (T_2_), 9 (T_3_), and 12 months (T_4_) after inclusion. Table 1 presents the types of data to be collected at the various intervals. The completed questionnaires are to be returned to the IQ Healthcare, Radboud University Nijmegen Medical Centre, by post, and the research assistant will maintain caregiver anonymity while processing the data. The researcher and the research assistant are not involved in the assignment procedure, and they do not know the assignment decision. The blinding of the outcome assessor must remain intact until follow-up measurements are completed. The research assistant will process the professionals' data after follow-up measurements are completed.

**Table 1:** Outcome measures

**Variable**	**PO**	**SO**	**EE**	**BG**	**Instrument/Source**	**T0**	**T1**	**T2**	**T3**	**T4**
**Professionals**										
Job satisfaction			✓		Job satisfaction, subscale from the Consultants' Mental Health Questionnaire	✓				✓
Gender				✓	SCPD questionnaire	✓				

**Informal caregivers**										
Sense of competence		✓			SCQ	✓				✓
Quality of life		✓			EQ-5D	✓				✓
Depressive symptoms		✓			CES-D	✓				✓
Caregiver distress		✓			NPI-Q	✓				✓
Time spent giving care			✓		RUD	✓	✓	✓	✓	✓
Age				✓	RUD	✓				
Gender				✓	RUD	✓				
Ethnicity				✓	RUD	✓				
Education				✓	RUD	✓				
Marital status				✓	RUD	✓				✓
Living arrangement				✓	RUD	✓				✓
Relation with the patient				✓	SCPD questionnaire	✓				
Shared household with the patient				✓	SCPD questionnaire	✓				✓

**People with dementia**										
Housing conditions/residence	✓		✓	✓	RUD	✓	✓	✓	✓	✓
Quality of life		✓			Qol-AD	✓				✓
Behavior problems		✓			NPI-Q	✓				✓
Healthcare services used			✓		RUD	✓	✓	✓	✓	✓
Severity of dementia				✓	DSM-III-TR	✓				
Age				✓	RUD	✓				
Gender				✓	RUD	✓				
Ethnicity				✓	RUD	✓				
Education				✓	RUD	✓				
Marital status				✓	RUD	✓				✓
Living arrangement				✓	RUD	✓				✓
Children				✓	RUD	✓				
Children living in				✓	RUD	✓				

PO, primary outcome; SO, secondary outcome; EE, economic evaluation; BG, background; T0, baseline measure; T1, 3-month follow-up measure; T2, 6-month follow-up measure; T3, 9-month follow-up measure; T4, 12-month follow-up measure; CES-D, Center for Epidemiologic Studies Depression Scale; DSM-III-TR, Diagnostic and statistical manual of mental disorders, 3^rd ^text revision; EQ-5D, EuroQol-5D; NPI-Q, Neuropsychiatric Problems Inventory Questionnaire; Qol-AD, Quality of Life in Alzheimer's Disease; RUD, Resource Utilization in Dementia; SCQ, Sense of Competence Questionnaire; SPCD, Systematic Care Program for Dementia.

### Outcome parameters

Table 1 presents the types of data used for the outcome parameters:

1. The primary outcome measure is the institutionalizing of the person with dementia in a nursing home during the 12-month follow-up period. Both the institutionalization rate and the time to institutionalization will be taken into account. Possible institutionalization will be assessed every 3 months with one item of the Resource Utilization in Dementia questionnaire[42].

2. The secondary outcome measure is the quality of the caregiver's and the patient's lives. The quality of the caregiver's life will be measured with the Sense of Competence Questionnaire [35], the EuroQol-5D [43,44], the Center for Epidemiologic Studies Depression Scale [45,46], and the caregiver distress will be assessed with the Neuropsychiatric Inventory Questionnaire [47,48]. The patient's quality of life will be measured with the Neuropsychiatric Problems Inventory Questionnaire and the Quality of Life in Alzheimer's Disease [49,50].

Baseline variables as well as sociodemographic characteristics of both the patient and the informal caregiver are control variables. Sociodemographic characteristics are the severity of the patient's dementia according the DSM-III-TR [41], and the caregiver's relation to the patient and their living arrangements. The health service, gender, and job satisfaction [51,52] of the professional are also control variables.

Concerning the effect of the intervention on emotional functioning we expect positive changes on the secondary outcome quality of life – e.g. depressive symptoms, patients' problem behavior and caregiver distress as related to patients' problem behavior, sense of competence, and both caregiver and patient quality of life – at 12 months follow-up, in favor of caregivers and patients involved in the intervention group, as compared to controls.

### Process evaluation

A process analysis of the intervention will be carried out to gain insight into factors that might influence success or failure of the intervention [53,54]. This process analysis is a description of the actual exposure of both the professional and the caregiver to the SCPD as planned and the experience of the professionals with the SCPD. For this purpose, the following questions will be examined retrospectively:

1. Were professionals trained in the SCPD as planned?

2. Did the informal caregivers receive the care as planned?

3. What is the relationship between the results when the SCPD was carried out as planned and the outcome measures of institutionalization, time to institutionalization, and the quality of the caregivers' lives?

4. What are the professionals' obstacles and facilitators for carrying out the SCPD as planned?

A triangulation of methods and data collection will be used to guarantee the internal validation of the process evaluation [55,56]. First, content analysis [57,58] will be used to determine whether the intervention group have been trained as planned. For this purpose, the data collected during the study period about the participation of professionals in one or more parts of the training sessions will be scored. Second, content analysis of the starter packages and action lists returned to us will be used to determine whether the caregivers received care from the intervention group as planned. The 11 items of the SCPD screening tool must have a score of 100% before we can assume that the caregiver has received care from the professional as planned. Because it is not possible to directly deduce whether the care received differs from the care planned, at least two items on the action list should be scored to make is credible that the caregiver received the care planned. We will present the results of both content analyses in tables of frequencies with cross tabulation.

These results will be the main input for the third question of the process evaluation, "What is the relationship between the results when the SCPD was carried out as planned and the outcome measures of institutionalization, time to institutionalization, and the quality of the caregivers' lives?" In answering this question, the carrying out of the SCPD as planned will be summarized as the product of two scores, namely, the score with professionals trained as planned and the score with caregivers receiving the care as planned. Next, this score will be used as the input for multilevel logistic and multilevel linear regression analyses for the relationship of the carrying out of the SCPD as planned to the institutionalization, time to institutionalization, and the quality of the caregivers' lives. The gender of the professional will be treated as a control variable to adjust for the characteristics of the professional. All process evaluation analyses will be done with SPSS version 16.0 (SPSS, Chicago, Illinois) and MLwiN Version 2.0 (Centre for Multilevel Modelling, University of Bristol, Bristol, UK).

Semi-structured interviews will be used to explore professionals' obstacles and facilitators in carrying out the SCPD as planned. Three key informants allocated to the intervention group from each health service will be interviewed. Purposive sampling [59] will be used to select a varied group of professionals from each health service on the basis of scores pertaining to professionals who carried out the SCPD as planned. In this approach, seven levels for exploring obstacles and facilitators are recognized: the intervention itself, the innovation itself, the individual professional, the patient (e.g., the caregiver), the social context, the organizational context, and the economic and political context [60]. The records of the evaluation sessions with professionals as part of their SCPD training, as well as information collected from two pilot interviews with professionals, will be used as input for developing question sets at each level. The interviews will be audio-taped and transcribed. Two investigators will use the modified grounded theory approach to analyze them independently [61-63]. We assume that 18 interviews will be enough to reach the point of theoretical saturation. If not, the purposive sampling procedure will be repeated, and additional interviews will take place until no new information about the obstacles and facilitators appear. The software package SPSS version 16.0 will be used for the purposive sampling procedure, and Atlas.ti 5.2 will be used for the qualitative analysis.

### Economic evaluation

The economics will be evaluated in parallel to the trial, which is compatible with the design presented earlier. The purpose of this evaluation is to determine the potential efficiency of the SCPD in the ambulatory mental healthcare setting versus usual care for the caregivers, from a societal perspective. The economic evaluation will be based on the general principles of a cost-effectiveness analysis, and the outcome measures will be costs, time to institutionalization of the patient, and the quality-adjusted life years (QALYs). These outcome measures will be combined in two incremental cost-effectiveness ratios (ICERs): cost per QALY gained and cost per unit of time-to-institutionalization gained. We will build up an empirical estimate of the sampling distributions of both ICERs by resampling with replacement from the original data (i.e., bootstrapping). A cost-effectiveness acceptability curve will summarize the evidence in support of SCPD being cost-effective for all potential values of the willingness to pay for a QALY, or a unit of time-to-institutionalization. We will explore the impact of uncertainty surrounding deterministic parameters (such as cost prices) on the ICER by means of one-way sensitivity analyses on the range of extremes.

The cost analysis will consist of two main parts. In the first part, on the patient-caregiver dyad level, we will measure volumes of care prospectively, using the RUD questionnaire [42,64]. This instrument contains questions about the use of community care services, type of accommodation, the employment status of the person with a cognitive disorder and the primary informal caregiver, medical care, and informal care. Informal care-giving time will be categorized as a loss of production (friction-cost method) for an employed primary caregiver, and as a loss of leisure time in all other cases. The RUD instrument will be completed by the informal caregiver every 3 months from baseline to follow-up at 12 months. The time to institutionalization is the final event. In both treatment groups, SCPD and usual care, the patients who are still not institutionalized at the end of 12 months are considered as censored observations. The second part of the cost analysis consists of determining the cost prices for each volume of consumption in order to be able to multiply the volumes registered for each participating caregiver and each patient. The Dutch guidelines for cost analyses will be used [65]. If no guideline or standard prices are available for units of care/resources, we will determine real cost prices with the activity-based costing method [66,67].

The effect analysis will adhere to the design of a cluster, randomized, controlled trial. The relevant variables for the economic evaluation are the time to institutionalization and the quality of the caregiver's life. We will use QALYs computed with the trapezium rule for a cost-utility analysis of the two treatment groups. The time to institutionalization will be the final event, meaning that the quality of the caregiver's life will be researched until the time of the patient's institutionalization, with a maximum of 12 months. We will use the standard EQ-5D [43,44] classification system developed by the EuroQol Group for the overall quantification of health status as a single index (utilities). The EQ-5D is one of three widely used multi-attribute systems available to determine health state preferences (utilities). The arguments for choosing the EQ-5D are:

1. The five domains of the EQ-5D reflect aspects that are thought to be important for the population under consideration.

2. The system is relatively simple to administer.

3. The sensitivity of the instrument has proven satisfactory.

4. A reasonably sound algorithm has been published to compute utilities.

### Power calculations

The difference in the expected effect is based on previous research in which 14% of the patients in the intervention group and 28% in the control group were institutionalized [35,36]. We need 132 patient-caregiver dyads for each of the intervention and control groups to detect a 50% reduction in institutionalization rates with 80% power at the two-sided significance level of 0.05. We inflated this sample size with a design effect of 1.15 to 152 dyads for each group to allow for correlating dyads within the same cluster, assuming an average cluster size of four and an intracluster correlation coefficient of 0.05. Assuming a 25% dropout rate of patient-caregiver dyads, the study needed a final enrolment of 190 dyads in each group, so that at least 48 professionals needed to be randomized to each group.

### Statistical analyses

Analyses will be performed at the level of the caregiver and the patient with dementia. All available data will be analyzed on an intention-to-treat basis, i.e. patient-caregiver dyads will remain in the group to which they are assigned. Descriptive analysis will be used to examine baseline comparability of both the intervention and control groups for sociodemographic characteristics, outcome parameters, and control variables. We will calculate the effect of the SCPD on the primary outcome measure (the number of institutionalizations) with Fisher's exact test. We will use multilevel logistic regression analyses to correct for the design effect of clustering patient-caregiver dyads with the professionals (level 1), and professionals in health services (level 2). We will use Kaplan-Meier survival analysis to quantify the effect of the SCPD on the primary outcome measure, time to institutionalization; and a Cox proportional hazard model, to correct for control variables. Analysis of the effect on the primary outcome and subgroup-analyses – adding the stratifying factor as a covariate and an interaction term of the stratifying factor with treatment group to the models – will be performed for shared household, age, and gender of the caregiver. Random coefficient regression analyses will be used to examine the effect of the SCPD on the secondary outcome measure (the quality of the patient's and the caregiver's lives) and also to correct for the clustering effect of the design, namely, patient-caregiver dyads clustered with professionals (level 1) and professionals in health services (level 2). We will also perform per protocol analyses. Mean substitution will be used for missing values unless at least if two third of the other items of that particular scale was completed. The software SPSS 16.0 (SPSS, Chicago, Illinois) and MLwiN Version 2.0 (Centre for Multilevel Modelling, University of Bristol, Bristol, UK) will be used for all statistical analyses.

### Ethical principles

The Committee on Research Involving Human Subjects, Arnhem-Nijmegen Region, approved the study protocol on March 9, 2005. Participation in the study is voluntary. Written consent must be obtained from all participating informal caregivers (see the section *Informed consent procedure*). Informal caregivers must explicitly be informed about the fact that they can withdraw their consent any time, without any specific reason, and with no negative consequences with regard to healthcare treatment now or in the future. Patient-caregiver dyads who withdraw from the study will continue to receive treatment from the professional they are assigned to. If a patient is institutionalized, or has died, the caregiver will no longer be invited to follow-up appointments.

Professionals from the health services allocated to the control group will not receive the SCPD training during the trial. However, they will be offered such training after the trial. This means that they can enter the SCPD, but with a 12-month delay.

Names of patient-caregiver dyads and other confidential information will be treated with medical confidentiality, and data are always separated from the names of the patient-caregiver dyads. Each participant is identified in the database by a number and an identity code. These codes are available only to the investigators and the research assistant.

The target groups are people with dementia, their informal caregivers, caregiver organizations, Alzheimer societies, professional healthcare workers, researchers of dementia care, and policy makers. The results of and information about the SCPD will be disseminated in publications and presentations at scientific and professional conferences and directly to family caregivers in Alzheimer cafes. The health services will also spread the results through their regional contacts.

## Discussion

### Strengths

A novelty in the SCPD for the caregivers is the pro-active and systematic approach, which involves informal caregivers in the support trajectory of the health service from the enrollment of the patient. Informal caregivers are systematically screened for a broad range of possible caregiver problems.

The use of an effective program to diagnose and systematically manage problems of these caregivers might improve the efficiency of the healthcare. Support for the caregivers is very important because these caregivers have greater burdens than caregivers of other chronically ill people [3], and they are at a greater risk of depression [4-6]. The SCPD attempts to contribute to the quality of the caregiver's and patient's lives by strengthening the caregiver's ability and sense of competence and by reducing patient's behavioral problems. The early detection and prevention of caregiver burden and depression may contribute to good results.

It is relevant to focus on the caregiver's sense of competence from the healthcare economic viewpoint, since a sense of competence is an important determinant of delaying institutionalization of the patient [10]. A relatively small cost investment for caregiver support from the SCPD would delay institutionalization, which is a major source of costs in the management of dementia. This is one of the three areas of greatest healthcare costs [7].

Neither the pro-active elements nor the systematic elements of our study approach are usual in the management of dementia in the ambulatory mental healthcare setting. To our knowledge, there are no similar studies underway at this moment.

### Limitations

Although a strong study design was used, some design characteristics might interfere with the reliability and validity of future results.

First, two forms of inclusion bias may have occurred. The first is the services' method of recruiting professionals: professionals were free to decide whether they wanted to participate. This may mean that participating professionals are more interested in care for the caregivers than their average colleagues. They may be more motivated to learn, and they might perform better than their non-participating colleagues. It is possible that they already take better care of caregivers than their colleagues. In practice, however, this form of inclusion bias is limited because almost all the available professionals participated to generate the necessary number of professionals. The second form of inclusion bias concerns the willingness of patient-caregiver dyads to remain in the study until their end-point is reached. The informal caregiver's burden may be an influential predictor of their willingness to participate. It would be reasonable if caregivers with a great burden did not want to participate because they could not handle any more work. Analyses of caregiver nonparticipation might be useful.

Second, the possibility of contamination arising due to a change of contacts and a possible knowledge exchange between professionals in the intervention and control groups cannot theoretically be excluded. To overcome this problem in practice, any professionals allocated to the intervention group were emphatically asked to keep the study intervention secret and not to give information about the intervention to the professionals in the control group, other collogues, or field relations. To evaluate the success or failure of this request, the intervention group will be evaluated at each training session for such knowledge. They will be asked if they have been questioned about the training by colleagues and whether they were able to keep the secret.

Third, professionals were aware of the dissemination of a study about supporting caregivers of people with dementia beforehand, and participating professionals are fully aware of their allocation. This may be a source of performance bias because the control group may treat patient-caregiver dyads differently than they used to. However, verification of performance bias is difficult because the actual usual ambulatory mental healthcare is still a black box. File investigation might determine whether professionals treated patient-caregiver dyads differently before, during, and after the study period.

Fourth, from an ethical point of view the question arises whether informed consent should be obtained from participating professionals because randomization took place at this level. This topic will be discussed from the professionals' point of view during the process analysis of the obstacles to and facilitators of the professional's participation in the study.

Fifth, patient-caregiver dyads were recruited from the mental health services, not from other institutions such as the outpatient clinics, the memory clinic, or directly from general practice. Thus our sample may not be representative of all patient-caregiver dyads.

### Policy implications

If the SCPD proves be effective in the ambulatory mental healthcare setting, wider implementation might be recommended. In that case, the organization will be promoted on a national level to include the SCPD in usual care. Enhancement of the quality of the patient's and caregiver's lives and delaying or preventing institutionalization of the patient will benefit all the target groups. Generalization to other countries may be limited because there are substantial differences in the design of organizing and financing healthcare (including long-term care), the provision of both informal and formal care (e.g., various types of residential accommodation), and cultural preferences concerning institutionalization within and between countries [68-70].

## Competing interests

The authors declare that they have no competing interests.

## Authors' contributions

MVD, the principle investigator, designed the study, gave the first training sessions in the SCPD and its subsequent use, and helped write the manuscript. AS assisted in designing the study and training the professionals in the SCPD and its subsequent use. AS also acquired the data, wrote the first draft of the manuscript, and was responsible for the revisions. FV, RG, and HW participated in the design of the study and helped draft of the manuscript. EA helped design the study and gave advice on the economic evaluation. MG helped draft the manuscript. All authors read and approved the final manuscript.

## Pre-publication history

The pre-publication history for this paper can be accessed here:


